# Antibody-Mediated Neutralization of the Exotoxin Mycolactone, the Main Virulence Factor Produced by *Mycobacterium ulcerans*

**DOI:** 10.1371/journal.pntd.0004808

**Published:** 2016-06-28

**Authors:** Jean-Pierre Dangy, Nicole Scherr, Philipp Gersbach, Melanie N. Hug, Raphael Bieri, Claudio Bomio, Jun Li, Sylwia Huber, Karl-Heinz Altmann, Gerd Pluschke

**Affiliations:** 1 Swiss Tropical and Public Health Institute, Basel, Switzerland; 2 University of Basel, Basel, Switzerland; 3 Department of Chemistry and Applied Biosciences, Institute of Pharmaceutical Sciences, Swiss Federal Institute of Technology (ETH) Zürich, Zürich, Switzerland; 4 Roche Innovation Center, Chemical Biology, Basel, Switzerland; Fondation Raoul Follereau, FRANCE

## Abstract

**Background:**

Mycolactone, the macrolide exotoxin produced by *Mycobacterium ulcerans*, causes extensive tissue destruction by inducing apoptosis of host cells. In this study, we aimed at the production of antibodies that could neutralize the cytotoxic activities of mycolactone.

**Methodology/Principal Findings:**

Using the B cell hybridoma technology, we generated a series of monoclonal antibodies with specificity for mycolactone from spleen cells of mice immunized with the protein conjugate of a truncated synthetic mycolactone derivative. L929 fibroblasts were used as a model system to investigate whether these antibodies can inhibit the biological effects of mycolactone. By measuring the metabolic activity of the fibroblasts, we found that anti-mycolactone mAbs can completely neutralize the cytotoxic activity of mycolactone.

**Conclusions/Significance:**

The toxin neutralizing capacity of anti-mycolactone mAbs supports the concept of evaluating the macrolide toxin as vaccine target.

## Author Summary

Mycolactones A/B (also simply referred to as mycolactone) are macrolide exotoxins produced by *Mycobacterium ulcerans*, the causative agent of the neglected tropical skin disease Buruli ulcer (BU). The potent cytotoxic and anti-inflammatory activities of mycolactones lead to severe destruction of the subcutaneous fat tissue with minimal inflammation in the core of the lesion. Due to the lipid-like nature of mycolactones, the production of antibodies against these molecules has so far been unsuccessful. By using the classical approach of fusing immortal cells with spleen cells of mice immunized with a protein conjugate of a truncated non-toxic synthetic mycolactone, we were able to generate hybridoma cells producing mycolactone-specific monoclonal antibodies. Mammalian cell-based cytotoxicity assays demonstrated that these antibodies have neutralizing activity and can fully block the cytotoxic activity of mycolactone, resulting in survival of the target cells. These findings support the concept to target mycolactone by a vaccine. Furthermore, the anti-mycolactone antibodies may represent useful tools for BU diagnostics development.

## Introduction

*Mycobacterium ulcerans*, the causative agent of the neglected tropical skin disease Buruli ulcer (BU), produces the exotoxin mycolactone, which is responsible for the formation of chronic necrotizing skin lesions [1, 2]. Early diagnosis followed by rapid initiation of the currently recommended treatment with rifampicin and streptomycin [3] is crucial to avoid massive tissue destruction and long-term disabilities.

Mycolactones consist of a 12-membered macrolide core structure, a short C-linked upper side chain (comprising C12–C20) and a longer C5-O-linked lower polyunsaturated acyl side chain. Mycolactones produced by different *M*. *ulcerans* lineages differ in the structure of the lower side chain, but are otherwise conserved [4]. For the lower side chain variations in length, the number of double bonds and the number and localization of hydroxyl groups have been described. While *M*. *ulcerans* strains may produce mixtures of several mycolactone species, the composition of these pools seems to be highly conserved for a particular *M*. *ulcerans* lineage [5]. Strains belonging to the genomically monomorphic classical African lineage produce the most toxic variant, mycolactone A/B [6]. This lineage is responsible for > 95% of the BU cases reported worldwide [7].

Mycolactone-deficient *M*. *ulcerans* mutants are less virulent and intradermal injection of the toxin in animal models is sufficient to induce the formation of BU-like lesions [4, 8]. Since mycolactones play such a central role in the pathogenesis of BU [1, 2, 9], they may represent a suitable target for both vaccine development and passive immunotherapy. However, due to their lipid-like nature, as well as their cytotoxic and immunosuppressive [10–12] activities, attempts to raise antibody responses against mycolactones have failed so far. While extracts from *M*. *ulcerans* cultures have been the only source of mycolactones for a long time, highly defined synthetic mycolactones have become available more recently [6, 13, 14], greatly facilitating experimental work with these compounds. Here, we generated mycolactone specific immune-sera and monoclonal antibodies (mAbs) by immunizing mice with a protein conjugate of a non-toxic synthetic truncated mycolactone derivative.

## Materials and Methods

### Ethics statement

Animal experiments performed were approved by the animal welfare committee of the Canton of Basel (authorization number 2375) and were conducted in compliance with the Swiss Animal Welfare Act (TSchG), Animal Welfare Ordinance (TSchV), and the Animal Experimentation Ordinance (TVV).

### Preparation of mycolactone stock solutions

Natural mycolactones A/B, C and F as well as variants PG-157, PG-165, PG-182 and core PG-119 were produced as described previously [6, 14]. The synthesis of biotin-conjugate PG-204 and the immunogen PG-203 will be described elsewhere. All compounds were HPLC-purified. For biological testing 0.5 mg/ml mycolactone stock solutions were made by adding cell culture grade DMSO (Sigma).

Extracts of mycolactone were prepared using mycobacterial pellets from cultures of strains isolated from lesions of Cameroonian BU patients (S1013, S1019, S1047). Briefly, 1 ml of a chloroform-methanol solution (2:1, v/v) was added to the individual pellets. Bacteria were resuspended by vortexing and lipids were extracted by incubating the samples shaking at RT for 2 h. Then, 200 μL ddH_2_O were added to induce a phase separation. After vigorous vortexing, the samples were centrifuged for 10 min and the lower organic phase was transferred to a fresh tube. Samples were placed in a Speedvac (Thermo Scientific) for complete drying. 50 μL acetone were added and after an additional spin for 10 min at maximal speed, the acetone-soluble lipid fraction containing mycolactones was separated from the precipitate, collected in fresh tubes and again dried using the Speedvac device. For biological testing, the acetone-soluble lipid fractions were resuspended in cell culture grade DMSO (Sigma).

### Immunization of mice

2 mg of mycolactone PG-203 were coupled to 2 mg of BSA using the Imject EDC BSA spin kit (Pierce). NMRI mice (Harlan Laboratories) were immunized thrice in 3 week intervals by subcutaneous injection of 40 μg of the coupled product emulsified in Sigma adjuvant. Serum antibody titers against the biotinylated mycolactone derivative PG-204 were tested by ELISA. Based on the ELISA results, one NMRI mouse was selected to receive a final intravenous injection of 40 μg of the PG-203-BSA conjugate without adjuvant. Three days after this last booster dose, hybridoma cell lines were generated as described below.

### Generation of monoclonal antibodies

Mice were euthanized and the spleen was aseptically removed. Spleen tissue was mashed and the cells were fused with PAI myeloma cells in the presence of PEG 1500 (Roche) [15]. Mother cell line culture supernatants were tested by ELISA for the presence of anti-mycolactone antibodies and positive lines were cloned by limiting dilution. mAbs were purified from hybridoma culture supernatants by affinity chromatography using a HiTrap Protein A HP column (GE Healthcare). Isotypes were determined by ELISA with isotype specific reagents (SouthernBiotech).

### ELISA

NeutrAvidin Coated High Capacity plates (Thermo Scientific) were incubated with 2 μg/ml biotinylated mycolactone PG-204 in PBS for 2 h at 37°C in the dark. The PG-204 solution was then replaced by SuperBlock T20 (TBS) blocking buffer (Thermo Scientific). Plates were incubated for 1 h at 37°C in the dark, then washed twice with PBS 0.05% Tween. Hybridoma supernatants were applied to the plates for 2 h at 37°C in the dark. Plates were washed four times with PBS 0.05% Tween. Bound anti-mycolactone antibodies were detected using an Alkaline Phosphatase (AP) conjugated goat anti-mouse IgG antibody (Sigma) diluted 1:20,000 in blocking buffer. The plate was incubated for 1 h at 37°C in the dark, and then washed four times with PBS 0.05% Tween. Development was done using the Alkaline Phosphatase Yellow (pNPP) Liquid Substrate System (Sigma). The absorbance was measured at 405 nm with a microplate reader (Tecan Sunrise) and the values were plotted against the concentration.

### Competition ELISA

For competition experiments Maxisorp plates were coated with mAbs (10 μg/ml) o/n and then blocked with SuperBlock T20 (TBS) blocking buffer (Thermo Scientific) for 1 h at 37°C. After washing twice with PBS 0.05% Tween, serial dilutions of synthetic mycolactone compounds were added and incubated for 2 h at 37°C in the dark. DMSO served as negative control. Subsequently biotinylated PG-204 was added (100 pg/ml) without washing steps and incubated for an additional 30 min. Plates were washed four times with PBS 0.05% Tween. Bound PG-204 was detected with alkaline phosphatase coupled streptavidin (SouthernBiotech) after 45 min incubation at 37°C. The development was performed as described above.

### Metabolic activity assays

Murine L929 fibroblasts were cultivated at 37°C and 5% CO_2_ in RPMI medium (Gibco) supplemented with 10% FCS (Sigma), 2 mM glutamine (Gibco) and 0.05 mM β-mercaptoethanol (Gibco). For the assay, L929 cells (24,000 per well) were seeded in 24-well plates (Falcon) and incubated o/n at 37°C. Medium was aliquoted and mixed with 15 ng/ml synthetic mycolactone A/B, mycolactone core PG-119 or varying amounts of mycolactone extracts. Dependent on the assay format, fixed or increasing concentrations of anti-mycolactone antibody or isotype-matching control antibody (JD4.1) were added and incubated for 10 min. The medium in the 24-well plates was aspirated and replaced by 500 μl medium containing the mycolactone-antibody mixes. After an incubation period of 48 h, Resazurin solution (Sigma) was added to the wells (10% v/v), and the cells were further incubated for 2 hours at 37°C and 5% CO_2_. In order to quantitatively assess metabolic activities, the fluorescence intensities were measured using a SpectraMax Gemini XS (Molecular Devices), and the obtained values were normalized for the DMSO control. The experiments were set up in duplicates and performed at least three times. Fluorescence intensities were plotted against the log concentration of neutralizing antibody.

### Surface Plasmon Resonance (SPR) analyses

SPR experiments were performed on Biacore 3000 or T200 instruments (GE Healthcare, Uppsala, Sweden) at 25°C. HEPES buffer (10 mM HEPES, 150 mM NaCl, 0.05% (w/v) Tween 20, pH 7.4) was used as running buffer and flow rates of 5 or 50 μl min^-1^ were used for the immobilization and binding assays, respectively. Anti-mycolactone mAbs were captured on the chip surface by primary antibodies. First, the primary antibody was immobilized covalently on the carboxymethyldextran surface of a CM5 sensor (GE Healthcare, Uppsala, Sweden). The carboxymethyl groups of the dextran sensor surface were activated for 600 s with a mixture of 0.2 M (3-dimethylaminopropyl)carbodiimide (EDC) and 0.05 M N-hydroxysuccinimide (NHS). Next, the primary antibody, a rat anti-mouse IgG1 mAb (SouthernBiotech, AL, USA) diluted in 10 mM borate buffer (pH 8.2) to a concentration of 20 μg/ml was contacted for 120 s with the activated sensor surface to achieve antibody densities of 8000–12000 RUs. Subsequently, remaining active ester molecules on the sensor were deactivated by applying 1M ethanolamine (pH 8.0) for 420 s. Anti-mycolactone mAbs were diluted in running buffer to a concentration of 200 nM and captured by the primary antibodies to achieve antibody densities in the range of 2500–4000 RUs.

Mycolactone derivative PG-204 was titrated over the surfaces with the immobilized anti-mycolactone mAbs. Association and dissociation phases were monitored for 240 and 3600 s, respectively. Bound mycolactone was washed out from the surface in regeneration steps (injection of 10 mM glycine, pH 2.2).

## Results

### Generation of mycolactone-specific mAbs

**Fig 1:**
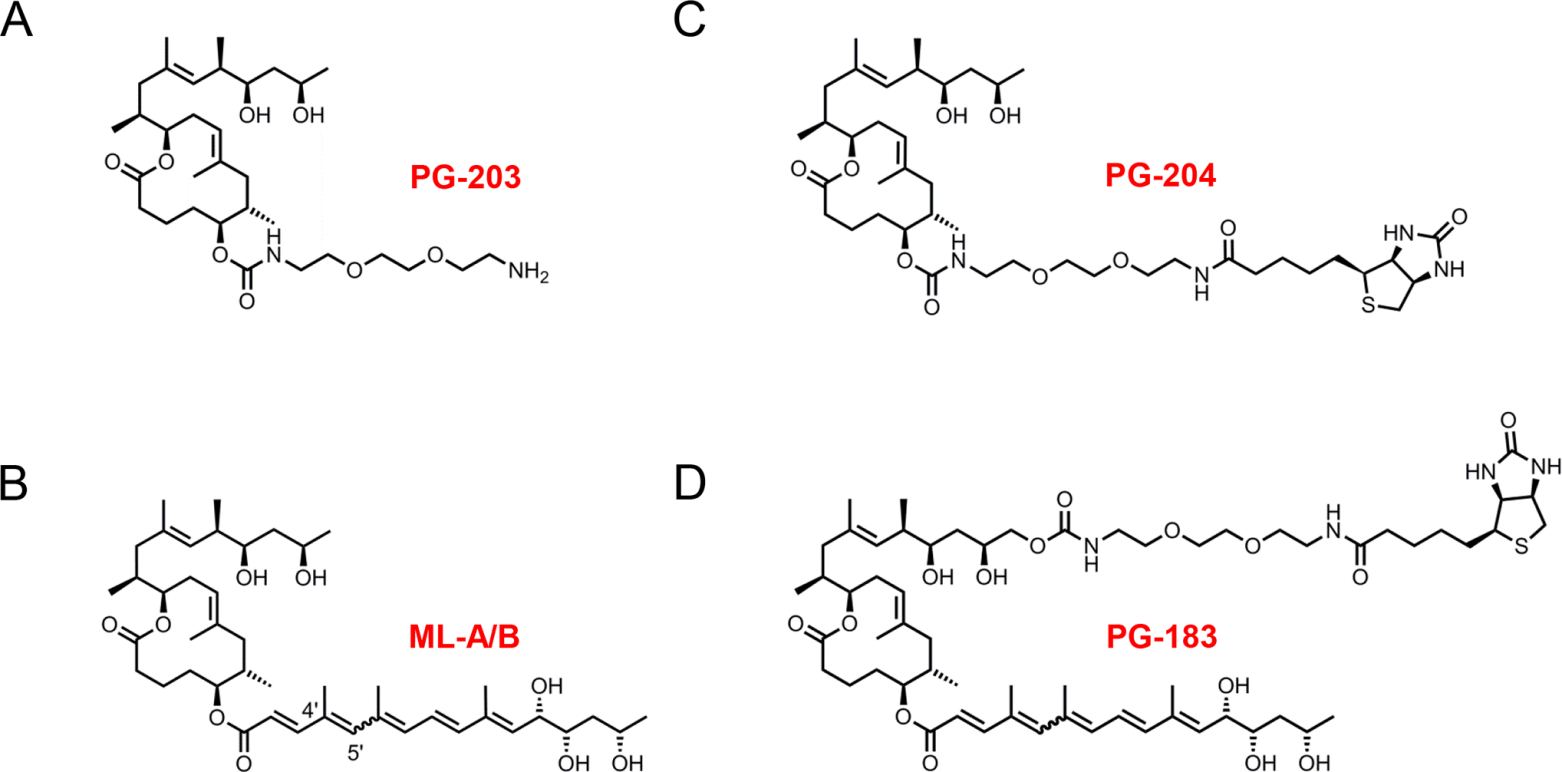
Synthetic mycolactone derivatives used for the generation and detection of mycolactone specific antibody responses. PG-203 (A) was conjugated to BSA via the amino group on its diethylene glycol-based linker and the carrier protein conjugate was used for the immunization of mice. PG-204 (C) and PG-183 (D) are biotinylated mycolactone derivatives used for analytical purposes. Unmodified synthetic mycolactone A/B (B) was used as effector molecule in cytotoxicity assays.

For the generation of mycolactone specific antibody responses we have immunized mice with PG-203, a truncated and non-cytotoxic mycolactone derivative coupled to BSA via a diethylene glycol-based linker replacing the C5-O-linked polyunsaturated acyl side chain (Fig 1A).

**Fig 2:**
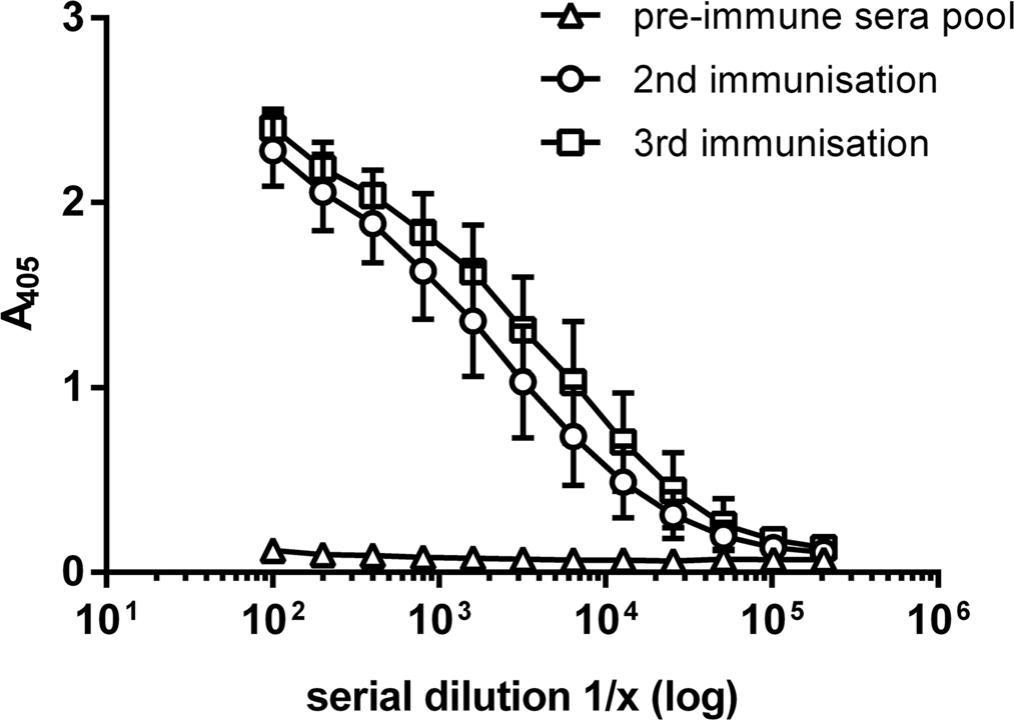
Mice immunized with the adjuvanted PG-203-BSA conjugate developed a boostable anti-mycolactone IgG response. Displayed are average anti-mycolactone IgG titers of three mice after the second and third immunization in comparison to pooled pre-immune sera. Titers were determined by ELISA using NeutrAvidin plates coated with PG-204.

Mice had developed strong anti-mycolactone IgG responses after two immunizations with the adjuvanted protein carrier conjugate and a third immunization boosted the immune response further (Fig 2).

**Fig 3:**
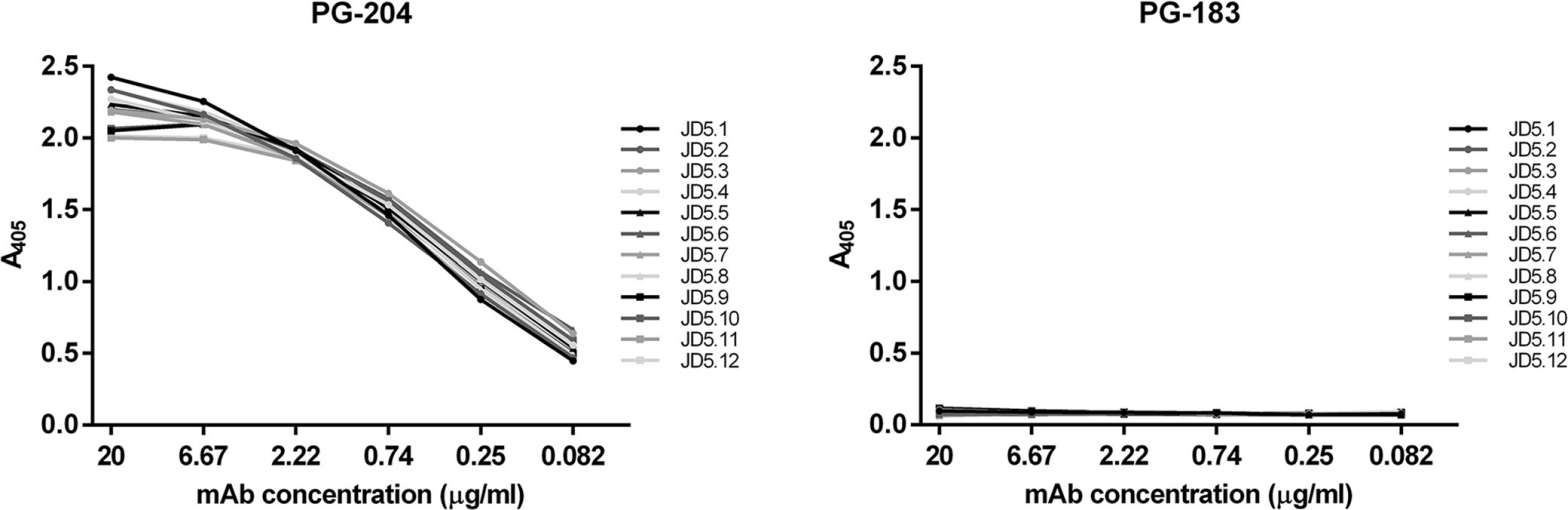
The anti-mycolactone mAbs JD5.1 to JD5.12 bind to PG-204, but not to PG-183. The biotinylated mycolactone derivatives PG-204 and PG-183 were bound to NeutrAvidin plates and incubated with serial dilutions of the mAbs. Bound anti-mycolactone mAbs were detected using alkaline phosphatase-conjugated goat anti-mouse IgG antibodies.

Spleen cells of mice immunized with the PG-203-BSA conjugate were used for the generation of hybridomas producing anti-mycolactone mAbs by conventional B cell hybridoma technology. Hybridoma cell culture supernatants were screened by ELISA using NeutrAvidin plates coated with PG-204 (Fig 1C), a biotinylated derivative of PG-203. Screening led to the identification of 12 hybridoma clones producing the anti-mycolactone mAbs JD5.1 to JD5.12. All twelve mAbs were of the IgG1 isotype and showed binding to PG-204, but not to PG-183 (Fig 3), a mycolactone derivative biotinylated at the C-linked short upper side chain (Fig 1D).

**Fig 4:**
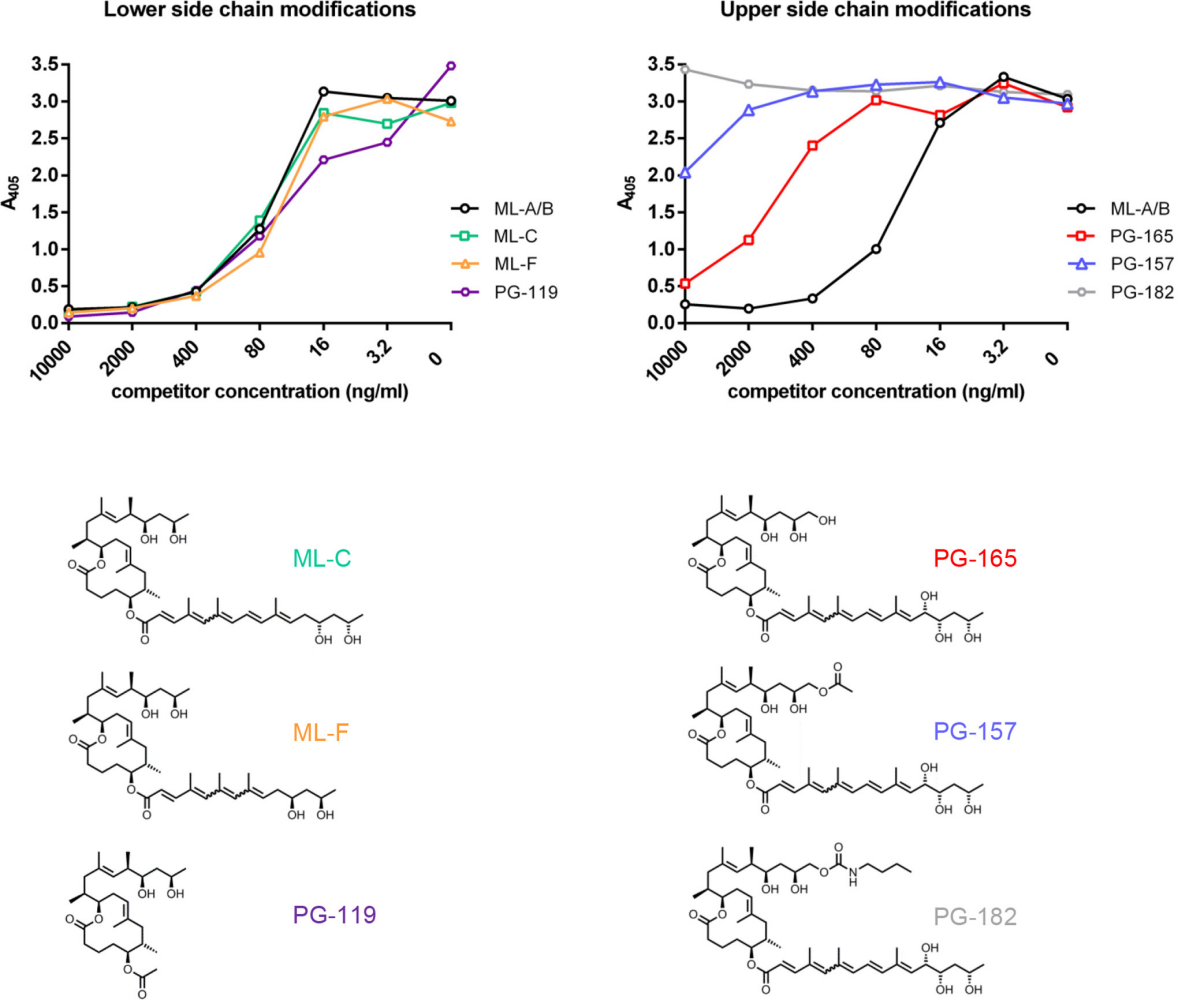
Effect of structural modifications of mycolactone on antibody binding. Competition ELISAs were performed by coating microtiter plates with the mAbs and pre-incubation with serial dilutions of mycolactone A/B, natural mycolactone variants harboring modifications in the lower fatty acid acyl side chain (mycolactone C and mycolactone F), a truncated core molecule without O-linked lower side chain (PG-119) or derivatives containing substitutions in the upper side chain (PG-165, PG-157 and PG-182). Biotinylated PG-204 was subsequently added and its binding was detected by using alkaline phosphatase-conjugated streptavidin as reporter. Detailed results with all tested mycolactone derivatives are shown for mAb 5.1 as example.

The fine specificity of mAbs was further characterized by competition ELISAs. Plates were coated with anti-mycolactone mAbs and the biotinylated mycolactone derivative PG-204 was added as reporter molecule. By titrating in increasing amounts of synthetic natural variants of mycolactone (mycolactone A/B, C and F), a truncated synthetic derivative (PG-119), or derivatives with modifications in the C-linked upper side chain (PG-157, PG-165 and PG-182) we were able to assess the fine specificity of the binding. All mAbs showed very similar fine specificity patterns (depicted for the prototype mAb JD5.1 in Fig 4). As predicted from the structure of the immunizing truncated mycolactone derivative PG-203, the presence/absence or detailed structure of the C5-O-linked polyunsaturated lower acyl side chain had no significant effect on binding to the mAbs. PG-119, a truncated mycolactone lacking the lower fatty acid acyl side chain was thus as efficient as the complete mycolactones A/B, C and F in competing with the reporter molecule PG-204 (Fig 4).

In contrast, modifications in the C-linked upper side chain had major effects on the efficiency as competitor. The more complex the modifications were, the less competition was observed (Fig 4), suggesting that the upper side chain along with the mycolactone core constitutes part of the epitope of the mAbs.

**Fig 5:**
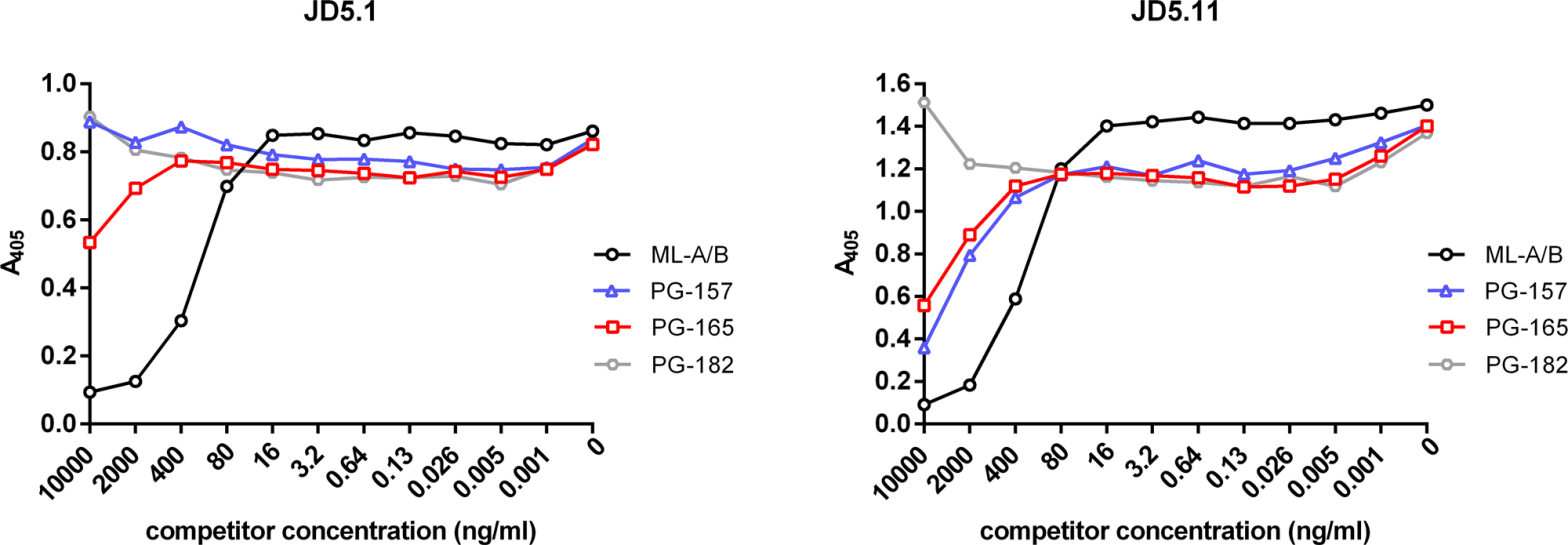
Two types of reactivity patterns. All mAbs displayed a similar fine specificity pattern, except for mAbs 5.9 and 5.11, which, in contrast to the other mAbs, showed inhibition with PG-157, a derivative with a slightly extended upper side chain (the difference is depicted for the prototype mAbs JD5.1 and JD5.11).

For all mAbs, addition of a terminal hydroxyl group in PG-165 reduced binding and a major extension of the upper side chain in PG-182 abrogated competition completely. Only for PG-157, a derivative with a slight extension of the upper side chain, a difference in the competition pattern was observed, with only mAbs 5.9 and 5.11 showing slight inhibition (the difference is depicted for the prototype mAbs JD5.1 and JD5.11 in Fig 5).

**Fig 6:**
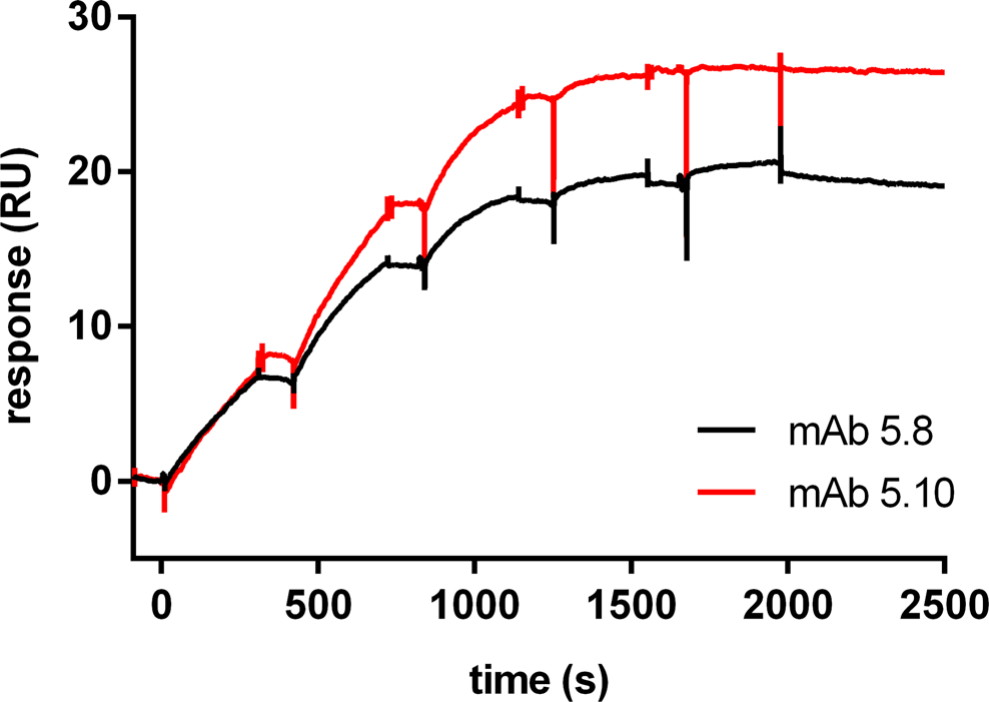
SPR analysis of anti-mycolactone mAbs. Anti-mycolactone mAbs were captured on the chip surface by primary antibodies. The biotinylated mycolactone derivative PG-204 was then titrated over the surface and association and dissociation phases were monitored for 240 and 3600 s, respectively. Prototype titration binding curves for two selected mAbs (JD5.8 and JD5.10) are shown. Titration of both antibodies was performed up to 500 nM with a dilution factor of 2.

In Surface Plasmon Resonance (SPR) analyses the anti-mycolactone mAbs immobilized on the surface of the sensor chip showed very tight binding to the biotinylated mycolactone derivative PG-204. The association and dissociation rate constants could not be precisely determined as the dissociation rate constants were outside the limitation of the measuring technology (Fig 6). For some of the mAbs (JD5.1, JD5.2, JD5.5, JD5.8 and JD5.10) dissociation rates were extremely slow with the monitored dissociation rate constant outside the limitation of the instrument resolution (k_off_ < 10^−6^ s^-1^). Also the other mAbs showed slow dissociation rates with an estimated k_off_ approximating the resolution limit (k_off_ between 10^−5^ s^-1^ and 10^−6^ s^-1^). Measurements with mycolactone A/B failed, most likely due to aggregation of the more hydrophobic complete toxin molecule.

### Toxin neutralizing activity of the anti-mycolactone mAbs

**Fig 7:**
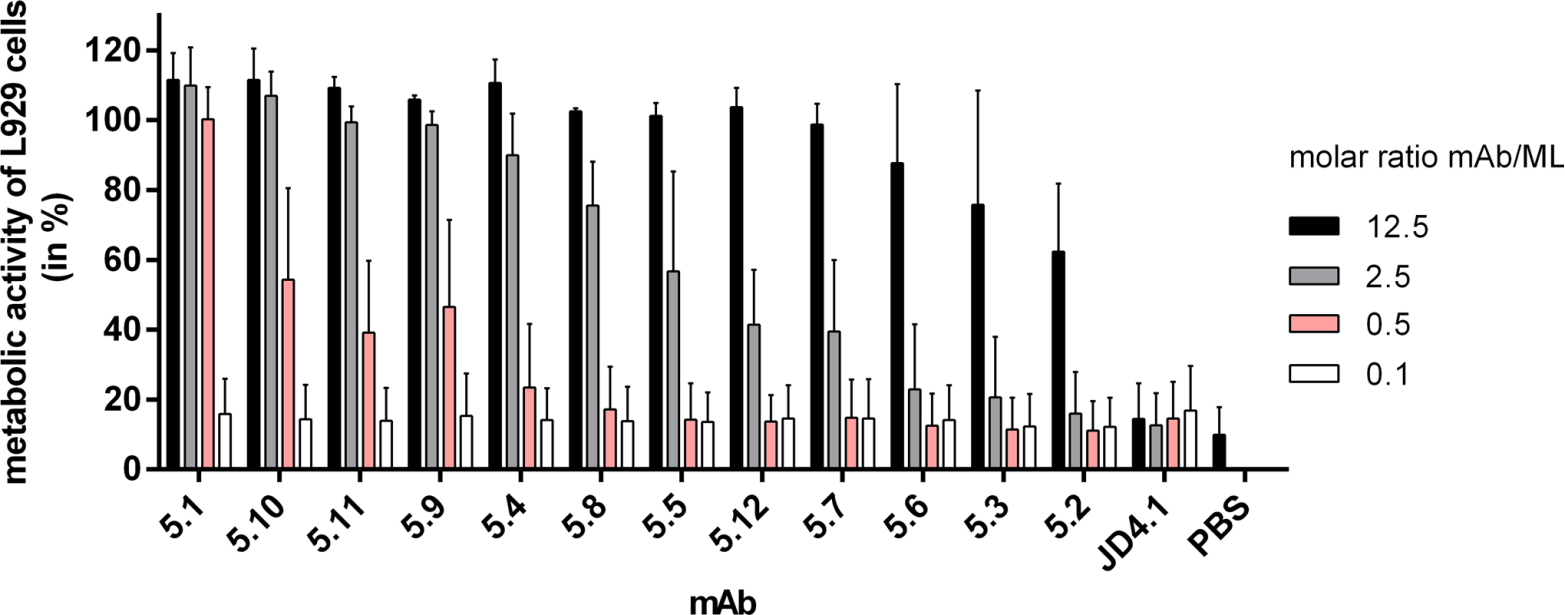
Toxin neutralizing activity of anti-mycolactone mAbs. L929 fibroblasts were incubated with 15 ng/ml (20 nM) synthetic mycolactone A/B and mAbs were added in different molar ratios of excess antibody versus mycolactone. After 48 h, the metabolic activity of fibroblasts was measured. The graph shows mean values across duplicate samples, and the error bars represent the standard deviation of three independent experiments. As control, the isotype-matched antibody JD4.1 was used.

To investigate whether mycolactone specific antibodies can neutralize the cytotoxic activity of the macrolide toxin, L929 fibroblasts were incubated with synthetic mycolactone A/B at the cytotoxic concentration of 15 ng/ml (20 nM) in the presence of serially diluted anti-mycolactone mAbs. After 48 h of incubation, the metabolic activity of the L929 cells was assessed by performing Resazurin-based assays. While all anti-mycolactone mAbs showed toxin-neutralizing activity, the molar ratio of antibody versus mycolactone required for neutralization varied (Fig 7). While several mAbs neutralized the toxin completely already at a molar ratio of 2.5, only partial inhibition was observed at a ratio of 12.5 for the least active mAb JD5.2 (Fig 7). The morphology of L929 cells treated with mycolactone and sufficient amounts of toxin-neutralizing antibody was indistinguishable from that of DMSO-treated control cells. In contrast, cells treated with no or insufficient amounts of antibody showed characteristic signs of apoptosis. The anti-mycolactone mAbs also neutralized the mycolactones produced by cultivated *M*. *ulcerans* bacteria (S1 Fig).

**Fig 8:**
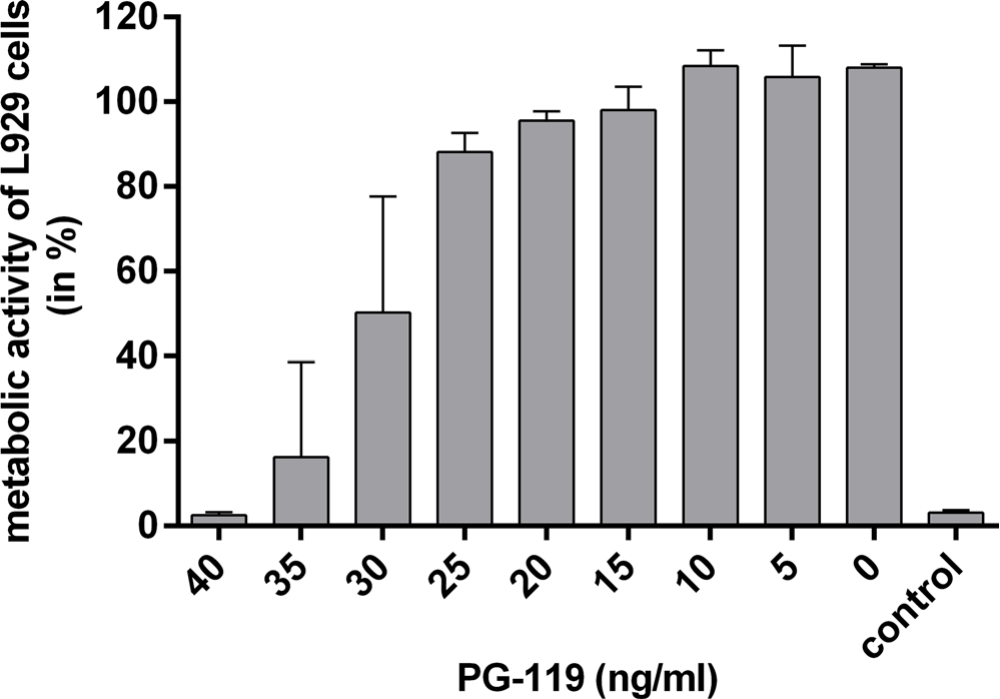
Inhibition of antibody mediated neutralization by addition of the non-toxic mycolactone core variant PG-119. L929 cells were treated with a mix of 15 ng/ml (20 nM) synthetic mycolactone A/B and a 2.5 fold molar excess of the anti-mycolactone mAb JD5.1. Serial dilutions of PG-119 were added to the mix, starting with a PG-119 concentration of 40 ng/ml (86 nM). As control, the isotype-matched antibody JD4.1 was used. After 48 h, the metabolic activity of fibroblasts was measured and plotted against the concentration of PG-119. The graph shows mean values across duplicate samples, and the error bars represent the standard deviation of three independent experiments.

Competition with the truncated non-toxic mycolactone derivative PG-119 lacking the lower acyl side chain (Fig 1) reconfirmed specificity of the neutralizing activity. At a concentration of 40 ng/ml (86 nM), PG-119 completely abrogated the toxin neutralizing activity of mAb JD5.1 (Fig 8).

## Discussion

Here we describe for the first time the production of antibodies against mycolactone, the main virulence factor of *M*. *ulcerans*. So far, no antibodies against mycolactone were detected in mice or humans infected with *M*. *ulcerans* [16] and attempts by several groups to generate mycolactone-specific antisera or mAbs by immunization with protein carrier conjugates of chemically modified mycolactone extracted from *M*. *ulcerans* cultures have failed before. This may be related to residual cytotoxicity of the conjugates and killing of B cells that incorporated them after binding to their mycolactone specific surface immunoglobulins. With the availability of synthetic mycolactone derivatives it has become possible to both generate highly defined protein conjugates for immunization and to develop reliable assays for the detection of mycolactone specific antibodies. For immunization we have used here a carrier conjugate of a mycolactone derivative in which the C5-O-linked polyunsaturated acyl side chain was replaced by a diethylene glycol-based linker. Since the structure of the lower side chain is crucially important for the cytotoxic activity of mycolactone [6], this compound (PG-203) was expected to be non-cytotoxic, even if it were released from the carrier protein after massive uptake by mycolactone specific B cells.

Coupling of the synthetic mycolactone derivative to the carrier protein BSA ensured T cell help for the mycolactone specific B cells, leading to clonal expansion, affinity maturation and isotype switching. As a result high anti-mycolactone IgG titers were elicited in mice already by two immunizations with the adjuvanted PG-203 carrier conjugate. All mAbs generated from the immunized mice were of the IgG1 subclass and exhibited high affinity and specificity for mycolactone. Competition assays indicated that the epitope recognized includes elements of the upper short side chain that is C-linked to the core structure. As expected [6], structural variation or absence of the lower C5-O-linked lower polyunsaturated acyl side chain had no effect on antibody binding. All mAbs exhibited high affinity binding with very slow dissociation rates which did not permit exact determination of the affinity constant by SPR analyses.

Currently, there is no highly effective vaccine against the major mycobacterial diseases tuberculosis, leprosy and BU available. BCG, originally developed against tuberculosis, may offer only partial and short-lasting [17] or no [18] protection against BU. Attempts to develop a subunit vaccine [16, 19, 20] or a live vaccine based on mycolactone-deficient *M*. *ulcerans* [21] had limited success. All twelve mycolactone-specific mAbs generated here showed, albeit to a varying degree, the capacity to neutralize mycolactone and to rescue mammalian cells from apoptosis in an *in vitro* assay. This supports the concept to target mycolactone in BU vaccine design. Both prophylaxis and therapy with toxin-neutralizing antibodies and active immunization with toxoids are highly successful strategies for protection against pathogens such as diphtheria or tetanus bacteria that produce a toxin as key virulence factor.

*M*. *ulcerans* has evolved from a common ancestor with *M*. *marinum* by acquisition of a plasmid designated pMUM, which encodes the polyketide synthases required for mycolactone biosynthesis [22–24]. None of the other pathogenic mycobacteria produce a macrolide toxin, making mycolactone an excellent target for the development of a species specific diagnostic test. Such an assay would also have potential for the monitoring of treatment success by measuring mycolactone levels in fine needle aspirates from closed BU lesions or swab samples from ulcerative lesions. The availability of the mAbs described here is enabling the development of a competition assay for the quantification of mycolactone. If non-competing mAbs specific for the lower part of the core and the lower side chain can be generated, development of an antigen capture assay may become possible.

Taken together, the first successful generation of mycolactone specific antibodies described in this report will stimulate development of new tools for research and control of BU.

## Supporting Information

S1 Fig Neutralization of mycolactone extracts by antibody JD5.1. From mycobacterial isolates S1013, S1019 and S1047, acetone-soluble lipids were extracted and added to L929 cells and incubated with mAb JD5.1. As control, the isotype-matched mAb JD4.1 was used. After 48 h, the metabolic activity of fibroblasts was measured. One representative of at least three independent experiments is shown.
(TIF)
