# Supplementary figures and images for: Antibody-Mediated Neutralization of the Exotoxin Mycolactone, the Main Virulence Factor Produced by Mycobacterium ulcerans
Source: PLoS Negl Trop Dis. 2016 Jun 28;10(6):e0004808. doi: 10.1371/journal.pntd.0004808 (PMC4924874; doi:10.1371/journal.pntd.0004808)

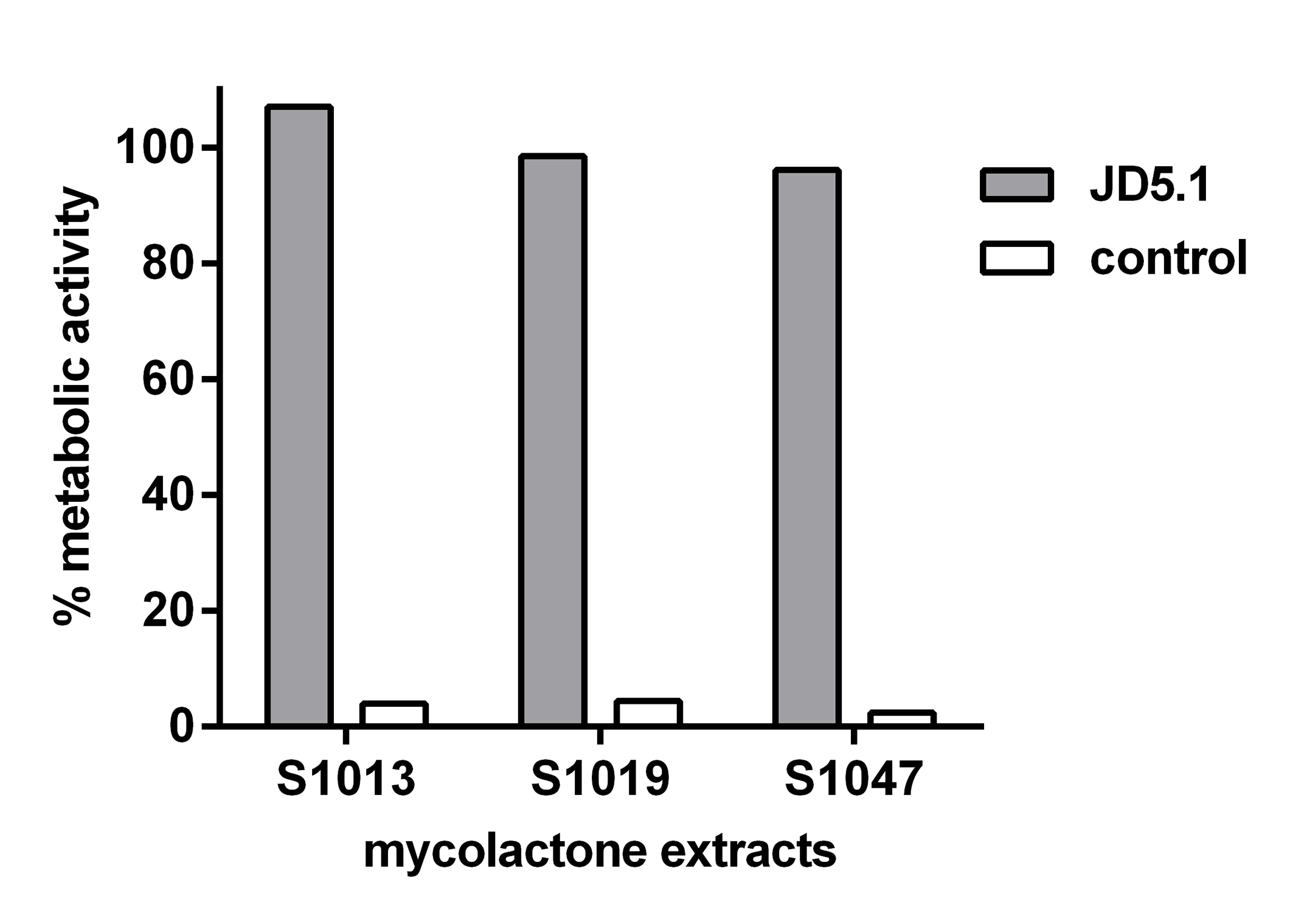

Supplement: S1 Fig — From mycobacterial isolates S1013, S1019 and S1047, acetone-soluble lipids were extracted and added to L929 cells and incubated with mAb JD5.1. As control, the isotype-matched mAb JD4.1 was used. After 48 h, the metabolic activity of fibroblasts was measured. One representative of at least three independent experiments is shown. (TIF) [file pntd.0004808.s001.tif]
